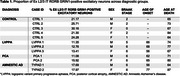# Validation of Ex L2/3 IT RORB GRIN1 Subpopulation Vulnerability in the Superior Temporal Gyrus Across Amnestic and Atypical Alzheimer's Disease Presentations

**DOI:** 10.1002/alz70861_108544

**Published:** 2025-12-23

**Authors:** Liara Rizzi, Ian Michael Oh, Song Hua Li, Felipe Luiz Pereira, Lea T. Grinberg

**Affiliations:** ^1^ Memory and Aging Center, UCSF Weill Institute for Neurosciences, University of California, San Francisco, San Francisco, CA USA; ^2^ Physiopathology in Aging Laboratory (LIM‐22), University of São Paulo Medical School, São Paulo, São Paulo Brazil; ^3^ Department of Pathology, University of São Paulo Medical School, São Paulo, São Paulo Brazil

## Abstract

**Background:**

Alzheimer’s disease (AD) is characterized by the selective vulnerability of specific neuronal populations. To investigate the molecular mechanisms underlying this susceptibility to AD‐related tau pathology, we previously performed single‐nucleus RNA sequencing (snRNA‐seq) on over 1.58 million nuclei and identified shared transcriptional alterations in excitatory neuron subtypes associated with AD pathology. Notably, the Ex L2/3 IT RORB GRIN1 subpopulation exhibited marked vulnerability in the Superior Temporal Gyrus (STG) in both amnestic and atypical AD presentations. In the present study, we aimed to validate the phenotype‐specific vulnerability of the Ex L2/3 IT RORB GRIN1 subpopulation to AD‐related tau pathology in excitatory neurons across amnestic AD, logopenic variant primary progressive aphasia (lvPPA), and posterior cortical atrophy (PCA) using quantitative neuropathological analysis.

**Method:**

Postmortem human STG brain tissue was sectioned into ∼6 μm paraffin slides from individuals with amnestic AD, lvPPA, PCA, and healthy controls. Immunohistochemistry was performed using markers for total neurons (NeuN), excitatory neurons (CAMKII), phospho‐tau pathology (PHF1), and the Ex L2/3 IT RORB GRIN1 subpopulation (BEX1). Fluorescent images were acquired using a ZEISS Axioscan 7 Slide Scanner, and the quantification of all markers was performed using ImageJ software.

**Result:**

In this initial validation, we analyzed samples from 5 healthy controls, 2 amnestic AD, 3 lvPPA, and 2 PCA cases. In each case, 1,000 neurons were analyzed. Among these, the number of excitatory neurons remained consistent across groups (772 ± 59.2), whereas the proportion of Ex L2/3 IT RORB GRIN1‐positive excitatory neurons was notably reduced in AD presentations. Approximately 24.5% of excitatory neurons in healthy controls expressed BEX1, compared to 17.8% in amnestic AD, 12.5% in lvPPA, and 13.5% in PCA cases. We are currently analyzing additional pathological cases to strengthen these findings.

**Conclusion:**

Our preliminary data suggests that the Ex L2/3 IT RORB GRIN1 subpopulation is depleted and selectively vulnerable to AD‐related tau pathology in excitatory neurons across both amnestic AD and atypical AD presentations. Ongoing analysis will further strengthen our findings. These results highlight the critical role of cell type–specific approaches in comprehending disease heterogeneity and progression.